# CD66b^+^ neutrophils and α‐SMA^+^ fibroblasts predict clinical outcomes and benefits from postoperative chemotherapy in gastric adenocarcinoma

**DOI:** 10.1002/cam4.2939

**Published:** 2020-02-25

**Authors:** Xiliang Cong, Yongle Zhang, Ziyu Zhu, Sen Li, Xin Yin, Zhao Zhai, Yu Zhang, Yingwei Xue

**Affiliations:** ^1^ Department of Gastrointestinal Surgery Harbin Medical University Cancer Hospital Harbin China; ^2^ Department of General Surgery The Affiliated Cancer Hospital of Zhengzhou University Zhengzhou China

**Keywords:** cancer‐associated fibroblasts, gastric adenocarcinoma, prognosis, tumor microenvironment, tumor‐associated neutrophils

## Abstract

**Background:**

Emerging evidence indicates that the tumor microenvironment (TME) influences tumor progression through the various cells it contains. Tumor‐associated neutrophils (TANs) and cancer‐associated fibroblasts (CAFs) are prominent constituents of diverse malignant solid tumors and are crucial in the TME and cancer evolution. However, the relationships and combined prognostic value of these two cell types are not known in gastric adenocarcinoma (GAC).

**Materials and Methods:**

In total, 215 GAC patients who underwent curative surgery were enrolled. TANs were assessed by immunohistochemical staining for CD66b, and CAFs were evaluated by immunohistochemical staining for α‐smooth muscle actin (α‐SMA).

**Results:**

The percentages of patients with high‐density TANs and CAFs in GAC tissue were 47.9% (103/215) and 43.3% (93/215), respectively. The densities of TANs and CAFs in GAC tissue samples were markedly elevated and independently correlated with GAC clinical outcomes. A strong correlation (*R* = .348, *P* < .001) was detected between TANs and CAFs in GAC. The combination of TANs and CAFs produced a more exact outcome than either factor alone. Patients with an α‐SMA^low^CD66b^high^ (hazard ratio [HR] = 1.791; 95% CI: 1.062‐3.021; *P* = .029), α‐SMA^high^CD66b^low^ (HR = 2.402; 95% CI: 1.379‐4.183; *P* = .002), or α‐SMA^high^CD66b^high^ (HR = 3.599; 95% CI: 2.330‐5.560; *P* < .001) phenotype were gradually correlated with poorer disease‐free survival than the subset of patients with an α‐SMA^low^CD66b^low^ phenotype. The same results were observed for disease‐specific survival in the subgroups. Noticeably, in stage II‐III patients with the α‐SMA^low^CD66b^low^ phenotype, an advantage was obtained with postoperative chemotherapeutics, and the risk of a poor prognosis was reduced compared with stage II‐III patients with the α‐SMA^low^CD66b^high^, α‐SMA^high^CD66b^low^ or α‐SMA^high^CD66b^high^ phenotype (HR: 0.260, 95% CI: 0.124‐0.542, *P* < .001 for disease‐free survival; and HR: 0.258, 95% CI: 124‐0.538, *P* < .001 for disease‐specific survival).

**Conclusion:**

Overall, we concluded that the combination of CD66b^+^ TANs and α‐SMA^+^ CAFs could be used as an independent factor for patient outcomes and to identify GAC patients who might benefit from the administration of postoperative chemotherapeutics.

## Graphical abstract

We concluded that the combination of CD66b^+^ tumor‐associated neutrophils and α‐SMA^+^ cancer‐associated fibroblasts could be used as an independent factor of outcome and to identify those gastric adenocarcinoma patients who might obtain advantage from postoperative chemotherapeutics.

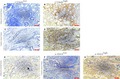

## 1 INTRODUCTION

Gastric adenocarcinoma (GAC) is the fifth most malignant tumor and the third leading cause of global cancer‐related mortality.^1^ Despite the development of multimodality treatment methods such as standard D2 lymphadenectomy surgery, systemic therapy, radiation therapy, and targeted treatments, the survival rate of GAC patients remains low.^2^, ^3^, ^4^ For patients with postoperative GAC, local recurrence and metastasis are considered major limitations, making adjuvant chemotherapy extremely critical.^5^ However, 85%‐90% of all gastric cancer patients respond poorly to adjuvant chemotherapy, and only a portion of patients achieve a stable condition or partial response to therapy.^6^ Hence, there is an urgent need to develop an exact prognostic tool that can be applied to reliably predict the risks of recurrence and metastasis and response to adjuvant chemotherapy in GAC patients. Currently, the TNM staging system is generally applied as a prognostic stratification tool by oncologists. However, the traditional TNM staging system provides only limited prognostic information and does not include information derived from the tumor microenvironment (TME). Thus, incorporating TME information with TNM staging might elevate the prognostic precision of the current model.

Gastric cancer is an inflammation‐associated tumor characterized by the invasion of multiple immune cells, including macrophagocytes, granular leukocytes, and different types of T lymphocytes.^7^, ^8^, ^9^ All these tumor‐related immune cells constitute a complex microenvironment that affects tumor development. Tumor‐associated neutrophils (TANs) are a predominant constituent of the inflammatory microenvironment and one of the predominant invasive immune cell populations in the tumor.^10^ Emerging evidence has indicated that TANs are pivotal for tumor initiation and progression.^11^, ^12^ TANs respond to signals from cancer cells or stromal cells by altering their phenotype and migratory pathways, and they also release factors that act on tumor cells.^13^, ^14^, ^15^ The increased frequency of TANs is associated with a poor prognosis in patients with solid tumors.^12^ TANs have been identified in several types of human tumors, including head and neck squamous cell carcinoma, gastric cancer, colorectal cancer, and renal cell carcinoma.^16^, ^17^, ^18^, ^19^ TANs secrete several soluble factors that cause carcinogenesis or accelerate cancer cell proliferation, cancer vasculogenesis, migration and invasion.^20^, ^21^, ^22^, ^23^ In addition, TANs also mediate tumor immune escape by inhibiting antitumor immunity.^24^

Cancer‐associated fibroblasts (CAFs) are spindle‐shaped fibroblast‐like interstitial cells expressing α‐smooth muscle actin (α‐SMA), constitute a primary fraction of the carcinoma stroma, and are frequently exposed to different inflammatory cells and mediators in the TME.^25^, ^26^ Thus, they may obtain new features that are not present in conventional fibroblasts, and these features tend to mediate reshaping of the TME and ultimately impact cancer evolution.^27^, ^28^, ^29^, ^30^ CAFs have an active role in mutual bidirectional interactions with tumor cells and other cell types in the TME, thereby promoting the niche that allows the tumor and promoting tumor development. Increasing studies have indicated that CAFs can be utilized as a significant prognostic marker in various tumors.^31^, ^32^, ^33^ Since TANs are the most common infiltrated type of immune cells in GAC, there may exist a forceful mutual effect between GAC‐derived CAFs and invasive TANs. In this research, we assessed the densities of TANs labeled with CD66b and CAFs labeled with α‐SMA in GAC by immunohistochemistry and focused on their combined effect on clinical outcomes, expecting to accurately predict patient prognosis and offer clues for stratified therapy in GAC patients.

## 2 MATERIAL AND METHODS

### 2.1 Patients

This retrospective study comprised 215 consecutive patients with GAC who underwent curative surgery with D2 lymphadenectomy at Harbin Medical University Cancer Hospital between January and December 2013. Inclusion criteria included pathologically confirmed adenocarcinoma, no preoperative chemotherapy and/or radiotherapy, accurate pathological TNM staging according to the 8th edition of the TNM classification of the American Joint Committee on Cancer staging manual, and integrated available follow‐up records. Patients who were lost to follow‐up, passed away during the perioperative period, had autoimmune diseases, with multiple cancers or previous cancers were excluded. Disease‐free survival (DFS) was calculated from the date of accepting surgery to the date of disease recurrence or metastases. Disease‐specific survival (DSS) was defined as the time between the date of accepting surgery and the date of death because of GAC. The histological subtypes were classified as well‐differentiated, moderately differentiated, and poorly differentiated GAC. Well and moderate differentiation include G1 and G2 GAC. Poor differentiation includes G3 GAC, gastric signet‐ring cell carcinoma, and mucinous GAC. The following clinicopathological parameters were collected for each patient from his/her medical records: sex, age, tumor size, tumor location, differentiation status, and pTNM stage. This research was approved by the ethics committee of Harbin Medical University Cancer Hospital.

### 2.2 Immunohistochemistry

Formalin‐fixed, paraffin‐embedded surgically resected tumor tissue samples were cut into 4‐μm sections. The sections were heated at 95°C for 20 minutes and dewaxed. Then, the slides were immersed in 3% H_2_O_2_ for 30 minutes to block endogenous peroxidase activity. The tissue sections were then incubated in a citrate buffer for 5 minutes on a 95‐99°C induction cooker for antigen retrieval and rinsed in phosphate‐buffered saline. Then, all sections were incubated in a humidified box at 4°C overnight with a monoclonal anti‐CD66b (555723, BD Biosciences, dilution 1:200) or primary polyclonal anti‐α‐SMA (55135‐1‐AP, Proteintech, dilution 1:200) antibody. Then, the specimens were incubated with secondary antibodies (SPN‐9001/2, anti‐rabbit/mouse IHC Kit, ZSGB‐BIO) for 1 hour at room temperature. Each slide was reacted with a 3‐3′‐diaminobenzidine reagent solution for 2 minutes and counterstained with hematoxylin for 20 seconds. Negative controls were processed in the same way without primary antibodies.

### 2.3 Assessment of immunostaining

Total immunohistochemical results and the CD66b‐ and α‐SMA‐positive cell densities were estimated by two independent gastroenterology pathologists (Chen K and Yan F) who were blinded to patient clinicopathological information. The number of CD66b‐positive neutrophils in each region was evaluated by applying Image‐Pro Plus 6.0 (Media Cybernetics). Uniform settings were applied to all images. Positive staining was determined in high‐power fields (HPFs, 200X). The intensity of neutrophil staining in histological sections was recorded as the average number of CD66b‐positive cells/HPF from 5 stochastic areas, and the mean was calculated. The median value was regarded as the threshold for low or high neutrophil density. α‐SMA immunoreactivity was evaluated as the percentage of positively stained cells and intensity of staining, which were scored as follows: (a) <5% colored cells as class 0, 5%‐25% as class 1, 26%‐50% as class 2, and >50% as class 3; and (b) no to weak staining intensity as class 0, moderate staining as class 1, and strong staining as class 2. The percentage and intensity scores were multiplied to form the low and high fibroblast density classes: 0‐2 indicated a low density, and higher than 2 indicated a high density.

### 2.4 Statistical analysis

All statistical analyses were performed using SPSS 22 (IBM Corp.). A two‐sided *P* < .05 was deemed significant. A paired‐sample t test was used to compare the CD66b^+^ TANs or α‐SMA^+^ CAFs in GAC specimens with those in matched paracarcinoma gastric tissue samples. The associations between quantitative variables were determined using the Pearson correlation coefficient. Chi‐square and Mann‐Whitney U tests were performed to evaluate associations between categorical variables. Kaplan‐Meier survival curves were constructed using the log‐rank test to assess DFS and DSS. Univariate and multivariate analyses using Cox proportional hazards models were applied to identify prognostic markers. Receiver operating characteristic (ROC) analysis was used to assess the value and accuracy of prognostic models.

## 3 RESULTS

### 3.1 TAN and CAF densities and their association in GAC patients

**Figure 1:**
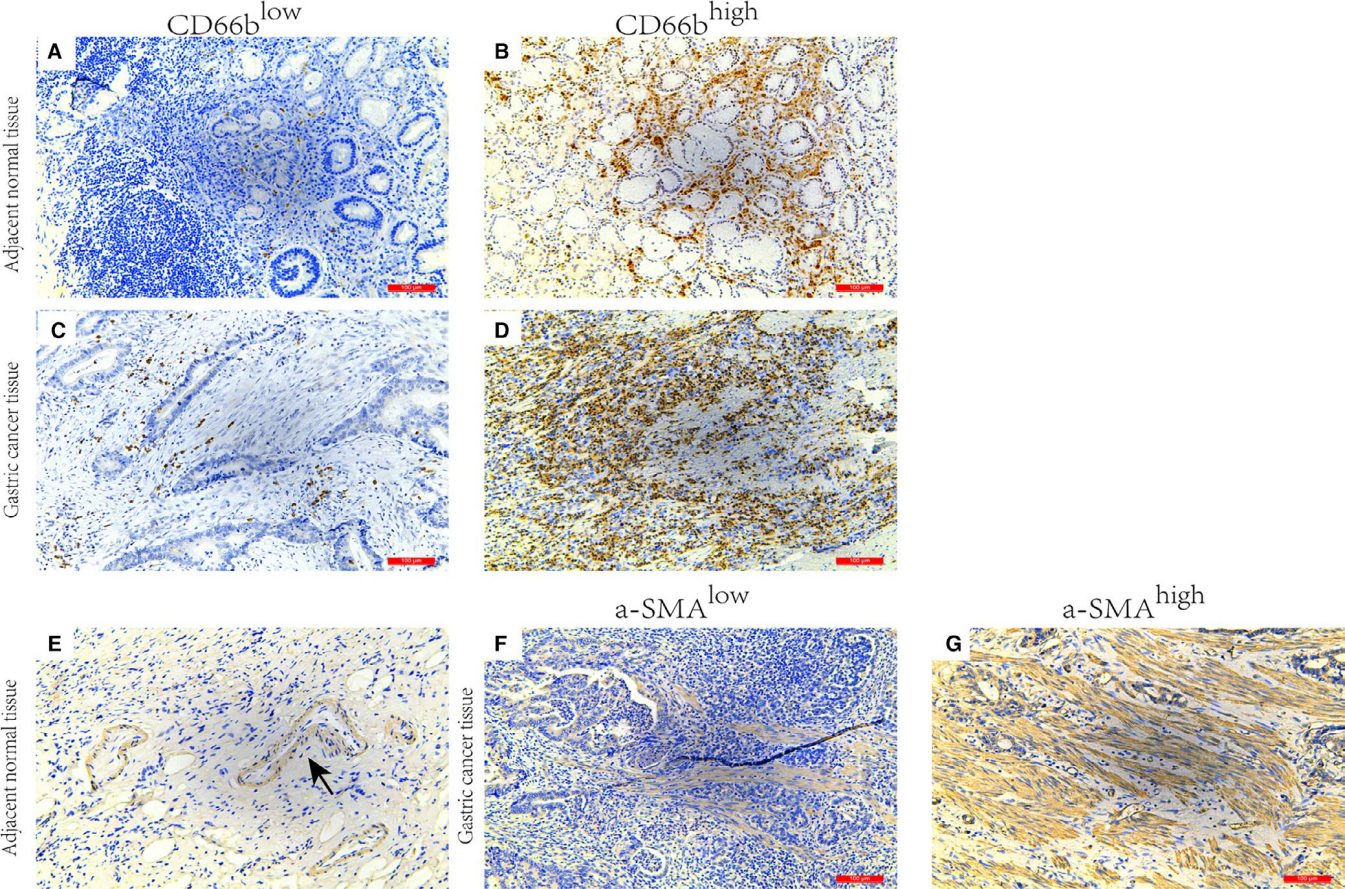
Immunohistochemical images of α‐SMA and CD66b expression in gastric adenocarcinoma tissue samples. Representative examples of low‐ and high‐density CD66b expression (A‐D) in gastric cancer tissue samples and adjacent normal tissue samples. Representative examples of low‐ and high‐density α‐SMA expression (E‐G) in gastric cancer tissue samples and adjacent normal tissue samples (arrow indicates positive α‐SMA staining of vascular smooth muscle cells). α‐SMA, α‐smooth muscle actin

**Figure 2:**
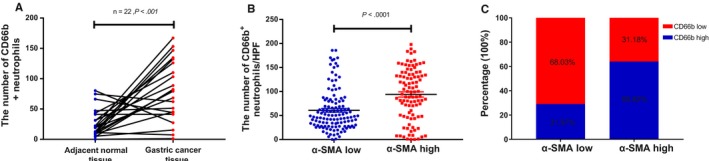
CD66b^+^ tumor‐associated neutrophil distribution in gastric cancer and the correlation with α‐SMA^+^ cancer‐associated fibroblasts. A. Assessed infiltration of CD66b^+^ tumor‐associated neutrophils in gastric cancer. B. Correlation between α‐SMA and CD66b expression in gastric cancer. C. CD66b^low^ and CD66b^high^ rates of patients in the α‐SMA groups. α‐SMA, α‐smooth muscle actin

In total, 215 patients were enrolled in this research. 107 of 185 patients at Ⅱ‐Ⅲ stage or Ⅰ stage with lymph node metastasis received fluorouracil‐based postoperative adjuvant chemotherapy (at least 1 cycle), mostly including capecitabine add platinum, capecitabine alone, or S‐1. The last follow‐up date was 31 August 2018. The overall follow‐up rate was 91.16% (196/215). 134 (62.3%) patients had verified recurrence after curative resection, and 128 (59.5%) had died at last follow‐up. The median follow‐up duration was 41.4 months. We performed immunohistochemical staining for CD66b and α‐SMA in GAC tissue samples. Representative images of CD66b^+^ and α‐SMA^+^ cells in the GAC tissue samples and matched normal tissue samples are shown in Figure 1. The results demonstrated that the density of CD66b^‐^positive cells in 22 GAC tissue samples was markedly higher than that in noncancerous matched specimens (Figure 2A, *P* < .001). The neutrophil distributions in the tumor tissue samples varied greatly among the GAC specimens, ranging from 0 to 198 cells/HPF. The CD66b^+^ staining indicated that TANs were distributed in a diffuse manner in the tumor stroma (Figure 1A‐D). Positive staining for α‐SMA in the nontumoral gastric tissue samples was recognized to identify vascular smooth muscle cells rather than stromal fibroblasts (Figure 1E). The expression of α‐SMA was identified in fibroblasts in the tumor stroma with no positive staining in GAC cells (Figure 1F‐G). The correlation between CD66b^+^ and α‐SMA^+^ cell densities was further evaluated. There was a significant positive correlation between the CD66b^+^ and α‐SMA^+^ cell densities (*R* = .384, *P* < .001) (Figure 2B). The rate of CD66b^+^ cells was remarkably higher in α‐SMA‐high GAC specimens than in α‐SMA‐low GAC specimens (68.82% vs. 31.18%, respectively; *P* < .001) (Figure 2C). None of the clinicopathological characteristics evaluated were associated with CD66b^+^ TANs (all *P* > .05). A high density of α‐SMA^+^ CAFs was prominently correlated with the late pTNM stage (Table 1).

**Table 1:** The correlations of α‐SMA and CD66b expression with clinicopathologic characteristics in gastric cancer patients

Characteristics	Total no, n	α‐SMA^+^CAFs, n (%)		*P* value	CD66b^+^TANs, n (%)		*P* value
		Low	High		Low	High	
All cases		122 (56.7)	93 (43.3)		112 (52.1)	103 (47.9)	
Sex				.167			.699
Female	59	29 (49.2)	30 (50.8)		32 (54.2)	27 (45.8)	
Male	156	93 (59.6)	63 (40.4)		80 (51.3)	76 (48.7)	
Age (years)				.902			.651
<60	103	58 (56.3)	45 (43.7)		52 (50.5)	51 (49.5)	
≥60	112	64 (57.1)	48 (42.9)		60 (53.6)	52 (46.4)	
Tumor size (cm)				.086			.102
<5	81	52 (64.2)	29 (35.8)		48 (59.3)	33 (40.7)	
≥5	134	70 (52.2)	64 (47.8)		64 (47.8)	70 (52.2)	
Differentiation				.374			.873
Well/moderate	47	24 (51.1)	23 (48.9)		24 (51.1)	23 (48.9)	
Poor	168	98 (58.3)	70 (41.7)		88 (52.4)	80 (47.6)	
Location				.161			.561
Upper	35	15 (42.9)	20 (57.1)		16 (45.7)	19 (54.3)	
Middle	45	25 (55.6)	20 (44.4)		22 (48.9)	23 (51.1)	
Lower	135	82 (60.7)	53 (39.3)		74 (54.8)	61 (45.2)	
pTNM stage				**.008**			.321
I	32	26 (81.3)	6 (18.8)		19 (59.4)	13 (40.6)	
II	48	23 (47.9)	25 (52.1)		28 (58.3)	20 (41.7)	
III	135	73 (54.1)	62 (45.9)		65 (48.1)	70 (51.9)	
Adjuvant chemotherapy				.411			.151
Yes	108	60 (54.1)	51 (45.9)		51 (47.2)	57 (52.8)	
No	107	62 (59.6)	42 (40.4)		61 (57.0)	46 (43.0)	

Abbreviations: α‐SMA, α‐smooth muscle actin; CAFs, cancer‐associated fibroblasts; TANs, tumor‐associated neutrophils.

*P* < .05 is considered statistically significant (bold).

### 3.2 Prognostic values of TANs and CAFs

**Figure 3:**
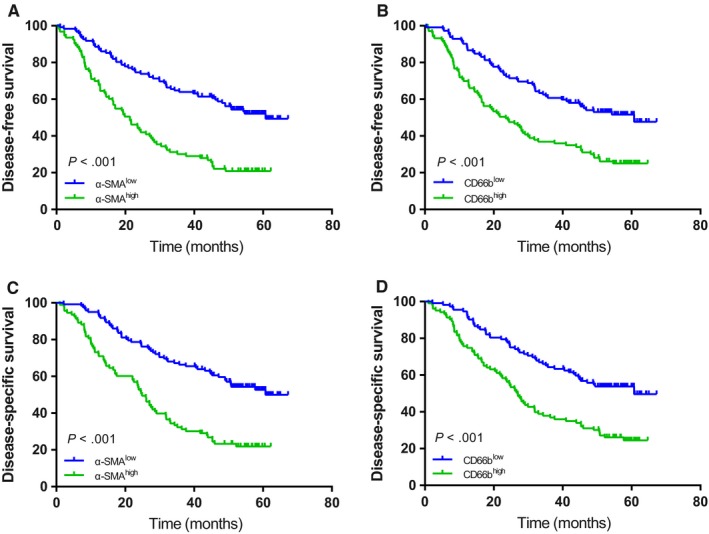
Kaplan‐Meier survival curves of patients with gastric cancer stratified according to α‐SMA and CD66b expression. DFS (A and B) and DSS (C and D) of patients with low and/or high densities of α‐SMA and CD66b in gastric cancer. α‐SMA, α‐smooth muscle actin; DFS, disease‐free survival; DSS, disease‐specific survival

**Figure 4:**
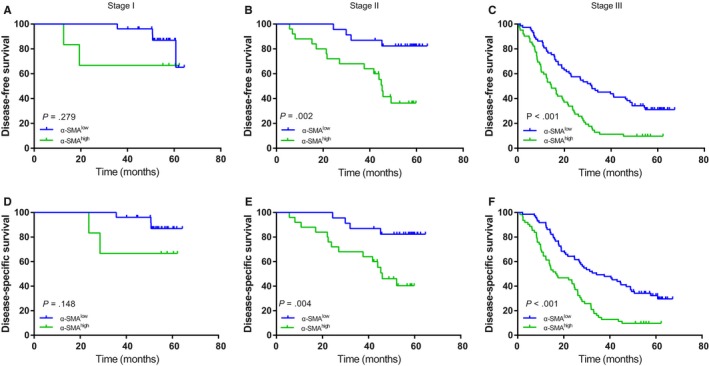
Kaplan‐Meier survival curves based on α‐SMA expression in gastric cancer patients (pTNM stage I‐III). DFS (A‐C) among subgroups stratified by α‐SMA expression. DSS (D‐F) among subgroups stratified by α‐SMA expression. α‐SMA, α‐smooth muscle actin; DFS, disease‐free survival; DSS, disease‐specific survival

**Figure 5:**
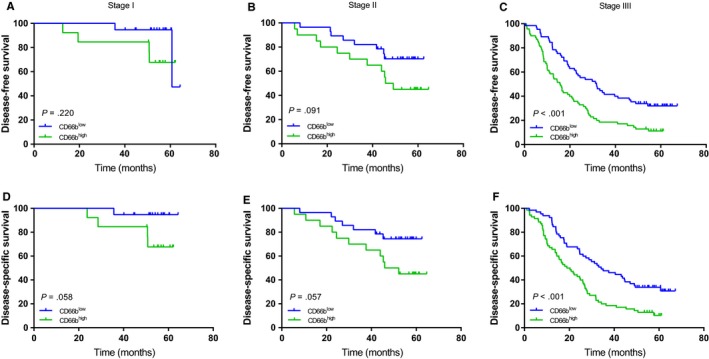
Kaplan‐Meier survival curves based on CD66b expression in gastric cancer patients (pTNM stage I‐III). DFS (A‐C) among subgroups stratified by CD66b expression. DSS (D‐F) among subgroups stratified by CD66b expression. DFS, disease‐free survival; DSS, disease‐specific survival

We conducted K‐M survival analyses and log‐rank tests to identify the prognostic diversity among patients categorized by the densities of TANs and CAFs. As shown in Figure 3, concomitant high densities of TANs and CAFs were related to compromised DFS and DSS (all *P* < .001). Compared to clinicopathological features, including tumor size, tumor location, adjuvant chemotherapy and pTNM stage, CD66^+^ (hazard ratio [HR] = 1.546; 95% CI = (1.055‐2.268); *P* = .026) and α‐SMA^+^ cells (HR = 2.212; 95% CI = 1.493‐3.278; *P* < .001) were independent factors for DFS in GAC. Similar results were acquired for these two factors for DSS (CD66b^+^: HR = 1.578, 95% CI = 1.072‐2.323, *P* = .021; α‐SMA^+^: HR = 2.278, 95% CI = 1.528‐3.395, *P* < .001). When pathological TNM stages (I‐III) were analyzed for patient stratification, survival curve analyses showed that a high density of α‐SMA^+^ CAFs predicted relatively poor DFS and DSS for stage II‐III GAC but not for stage I GAC (Figure 4). However, a high density of CD66^+^ TANs predicted relatively poor DFS and DSS in phase III GAC only (Figure 5).

### 3.3 Prognostic value of the combination of TANs and CAFs in GAC

**Figure 6:**
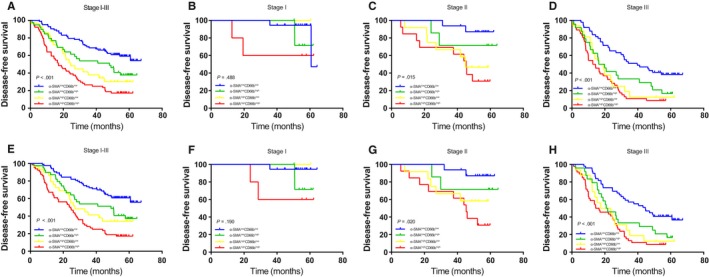
Kaplan‐Meier survival curves based on α‐SMA and CD66b coexpression in gastric cancer patients (pTNM stage I‐III). DFS (A‐D) among subgroups stratified by the combination of α‐SMA and CD66b expression. DSS (E‐H) among subgroups stratified by the combination of α‐SMA and CD66b expression. α‐SMA, α‐smooth muscle actin; DFS, disease‐free survival; DSS, disease‐specific survival

We divided 215 GAC patients into four subgroups according to their CD66b^+^ TAN and α‐SMA^+^ CAF densities as follows: α‐SMA^low^CD66b^low^ (n = 83, 38.6%), α‐SMA^low^CD66b^high^ (n = 39, 18.1%), α‐SMA^high^CD66b^low^ (n = 29, 13.5%), and α‐SMA^high^CD66b^high^ (n = 64, 29.8%). The patients with the α‐SMA^high^CD66b^high^ phenotype had the poorest DFS and DSS among all 4 subsets, while the patients with the α‐SMA^low^CD66b^low^ phenotype had the best DFS and DSS (Figure 6). pTNM stage (*P* = .021) was significantly correlated with patient subsets classified by the two‐marker categorizer, as shown in Table 2. Univariate Cox proportional hazard model analysis indicated that the α‐SMA^low^CD66b^high^ (HR = 1.791; 95% CI = 1.062‐3.021; *P* = .029), α‐SMA^high^CD66b^low^ (HR = 2.402; 95% CI = 1.379‐4.183; *P = *.002) and α‐SMA^high^CD66b^high^ (HR = 3.599; 95% CI = 2.330‐5.560; *P* < .001) patient subgroups were progressively correlated with poorer DFS compared with the α‐SMA^low^CD66b^low^ patient subgroup. In a multivariate Cox proportional hazard model, this two‐biomarker categorizer could independently predict the clinical outcome of GAC with progressively increasing hazard ratio values when the α‐SMA^low^CD66b^low^ subset was treated as a reference (Table 3).

**Table 2:** Associations of α‐SMA and CD66b coexpression with clinicopathologic characteristics in patients with gastric cancer

Characteristics	α‐SMA^+^CAFs & CD66b^+^TANs, n (%)				*P* value
	α‐SMA^low^CD66b^low^	α‐SMA^low^CD66b^high^	α‐SMA^high^CD66b^low^	α‐SMA^high^CD66b^high^	
All cases	83 (38.6)	39 (18.1)	29 (13.5)	64 (29.8)	
Sex					.265
Female	23 (39.0)	6 (10.2)	9 (15.3)	21 (35.6)	
Male	60 (38.5)	33 (21.2)	20 (12.8)	43 (27.6)	
Age (years)					.953
<60	38 (36.9)	20 (19.4)	14 (13.6)	31 (30.1)	
≥60	45 (40.2)	19 (17.0)	15 (13.4)	33 (29.5)	
Tumor size (cm)					.237
<5	38 (46.9)	14 (17.3)	10 (12.3)	19 (23.5)	
≥5	45 (33.6)	25 (18.7)	19 (14.2)	45 (33.6)	
Differentiation					.540
Well/moderate	15 (31.9)	9 (19.1)	9 (19.1)	14 (29.8)	
Poor	68 (40.5)	30 (17.9)	20 (11.9)	50 (29.8)	
Location					.353
Upper	12 (34.3)	3 (8.6)	4 (11.4)	16 (45.7)	
Middle	16 (35.6)	9 (20.0)	6 (13.3)	14 (31.1)	
Lower	55 (40.7)	27 (20.0)	19 (14.1)	34 (25.2)	
pTNM stage					**.021**
I	18 (56.3)	8 (25.0)	1 (3.1)	5 (15.6)	
II	16 (33.3)	7 (14.6)	12 (25.0)	13 (27.1)	
III	49 (36.3)	24 (17.8)	16 (11.9)	46 (34.1)	
Adjuvant chemotherapy					.496
Yes	39 (36.1)	21 (19.4)	12 (11.1)	36 (33.3)	
No	44 (41.1)	18 (16.8)	17 (15.9)	28 (26.2)	

Abbreviations: α‐SMA, α‐smooth muscle actin; CAFs, cancer‐associated fibroblasts; TANs, tumor‐associated neutrophils.

*P* < .05 is considered statistically significant (bold).

**Table 3:** Univariate and multivariate Cox regression analyses for DFS and DSS based on α‐SMA and CD66b coexpression stratification and clinicopathologic characteristics

Variables	Disease‐free survival				Disease‐specific survival			
	Univariate analysis		Multivariate analysis		Univariate analysis		Multivariate analysis	
	HR (95% CI)	*P* value	HR (95% CI)	*P* value	HR (95% CI)	P value	HR (95% CI)	*P* value
Sex		.337				.330		
Female	1				1			
Male	0.830 (0.567‐1.214)				0.827 (0.564‐1.212)			
Age (years)		.834				.596		
<60	1				1			
≥60	1.037 (0.736‐1.462)				1.098 (0.777‐1.553)			
Tumor size (cm)		**.002**		.272		**<.001**		.048
<5	1		1		1		1	
≥5	1.830 (1.257‐2.663)		1.245 (0.842‐1.840)		1.986 (1.355‐2.910)		1.492 (1.003‐2.221)	
Differentiation		.200				.163		
Well/moderate	1				1			
Poor	1.325 (0.862‐2.036)				1.365 (0.882‐2.113)			
Location		**.017**		.071		**.043**		.204
Upper	1		1		1		1	
Middle	0.803 (0.476‐1.353) 0.409		0.892 (0.522‐1.525)	.677	0.823 (0.484‐1.401)	.473	0.953 (0.551‐1.648)	.863
Lower	0.548 (0.350‐0.857) 0.008		0.626 (0.395‐0.993)	.047	0.586 (0.372‐0.923)	.021	0.706 (0.442‐1.128)	.145
pTNM stage		**<.001**		**<.001**		**<.001**		**<.001**
I	1		1		1		1	
II	2.435 (0.972‐6.098) 0.057	.057	2.030 (0.793‐5.199)	.140	2.788 (1.035‐7.509)	**.043**	2.242 (0.817‐6.150)	.117
III	7.860 (3.444‐17.938)	**<.001**	7.368 (3.152‐17.225)	**<.001**	9.293 (3.781‐22.840)	**<.001**	8.484 (3.391‐21.228)	**<.001**
Adjuvant chemotherapy		**.004**		**.001**		**.003**		**<.001**
Yes	1		1		1		1	
No	1.680 (1.183‐2.387)		1.931 (1.342‐2.780)		1.688 (1.191‐2.394)		2.054 (1.423‐2.966)	
α‐SMA^+^CAFs & CD66b^+^TANs		**<.001**		**<.001**		**<.001**		**<.001**
α‐SMA^low^CD66b^low^	1		1		1		1	
α‐SMA^low^CD66b^high^	1.791 (1.062‐3.021)	**.029**	1.900 (1.112‐3.244)	**.019**	1.835 (1.084‐3.106)	**.024**	1.923 (1.123‐3.294)	**.017**
α‐SMA^high^CD66b^low^	2.402 (1.379‐4.183)	**.002**	2.771 (1.574‐4.878)	**<.001**	2.363 (1.341‐4.163)	**.003**	2.848 (1.597‐5.082)	**<.001**
α‐SMA^high^CD66b^high^	3.599 (2.330‐5.560)	**<.001**	3.511 (2.254‐5.469)	**<.001**	3.660 (2.361‐5.676)	**<.001**	3.689 (2.357‐5.775)	**<.001**

Abbreviations: α‐SMA, α‐smooth muscle actin; CAFs, cancer‐associated fibroblasts; CI, confidence interval; DFS, disease‐free survival; DSS, disease‐specific survival; HR, hazard ratio; TANs, tumor‐associated neutrophils.

*P* < .05 is considered statistically significant (bold).

### 3.4 Extension and accuracy of prognostic models including the two‐marker predictor

**Figure 7:**
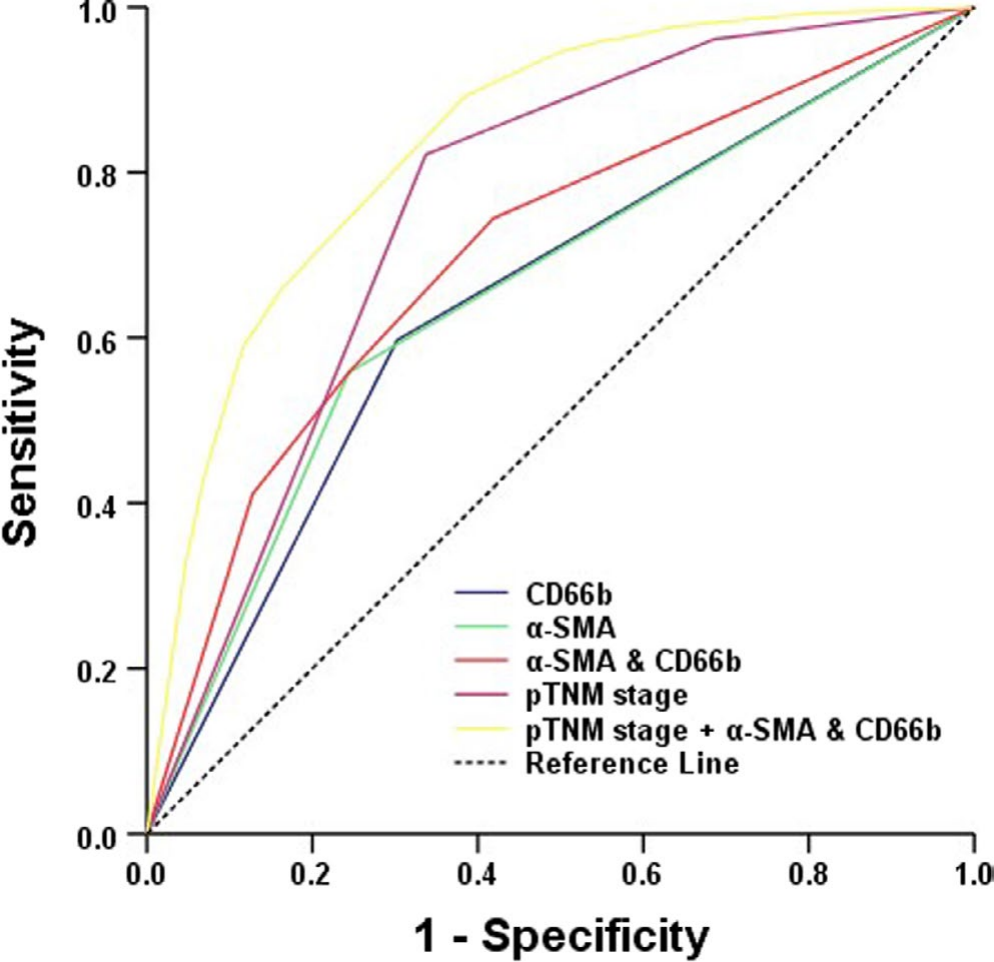
Receiver operating characteristic (ROC) curves for prognostic markers predicting survival among GAC patients. GAC, gastric adenocarcinoma

Considering this distinct prognostic value, we combined the two‐marker classifier with the pTNM staging system to investigate the actual prognostic value of the combination of TANs and CAFs. ROC analysis was applied, and area under the curve (AUC) values were compared to evaluate prognostic accuracy. The AUCs of TANs or CAFs alone were 0.647 and 0.657, respectively, while the combination of the two markers had an AUC of 0.703 (Figure 7). The AUC of pTNM staging alone was 0.757, and the AUC was elevated to 0.839 when the two‐marker predictor was added (Table 4).

**Table 4:** Areas under ROC curves for prognostic markers

Factor	AUC (95% CI)	*P*‐value
CD66b	0.647 (0.572‐0.722)	**<.001**
α‐SMA	0.657 (0.583‐0.731)	**<.001**
α‐SMA & CD66b	0.703 (0.633‐0.774)	**<.001**
pTNM stage	0.757 (0.688‐0.827)	**<.001**
pTNM stage + α‐SMA & CD66b	0.839 (0.784‐0.894)	**<.001**

Abbreviations: α‐SMA, α‐smooth muscle actin; AUC, area under the curve; CI, confidence interval; ROC, receiver operating characteristic.

*P* < .05 is considered statistically significant (bold).

### 3.5 Correlations between the two‐marker predictor and adjuvant chemotherapy

**Figure 8:**
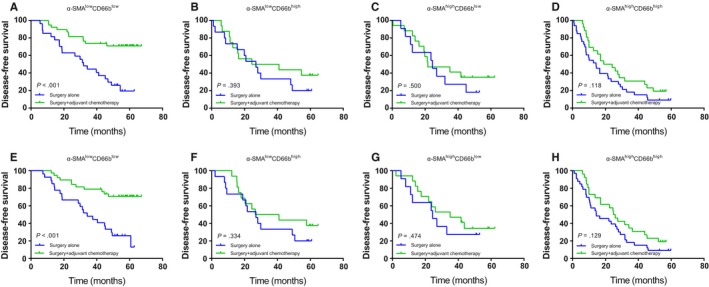
Kaplan‐Meier survival curves for gastric cancer patients who underwent surgery alone or surgery + adjuvant chemotherapy stratified according to α‐SMA and CD66b coexpression. DFS (A‐D) and DSS (E‐H) in α‐SMA^low^CD66b^low^, α‐SMA^low^CD66b^high^, α‐SMA^high^CD66b^low^, and α‐SMA^high^CD66b^high^ gastric cancer patients. α‐SMA, α‐smooth muscle actin; DFS, disease‐free survival; DSS, disease‐specific survival

In a subgroup analysis, we assessed the advantage of postoperative chemotherapeutics in TNM stage II‐III patients. There is only a weak relationship between patients with the α‐SMA^low^CD66b^high^, α‐SMA^high^CD66b^low^ or α‐SMA^high^CD66^high^ phenotype and survival (DFS: *P* = .393, *P* = .500 and *P* = .118, respectively, Figure 8B‐D; DSS: *P* = .334, *P* = .474, *P* = .129, respectively, Figure 8F‐H), and these make only a small contribution to the difference between patients with surgery only and with surgery add postoperative chemotherapy adjuvant in HR. However, the patients with the α‐SMA^low^CD66b^low^ phenotype could receive a significant benefit from adjuvant chemotherapy (DFS: *P* < .001, Figure 8A; DSS: *P* < .001, Figure 8E). Treatment with adjuvant chemotherapy was associated with a decreasing risk of a poor clinical outcome in the α‐SMA^low^CD66b^low^ patient subset (HR: 0.260, 95% CI: 0.124‐0.542, *P* < .001; HR: 0.258, 95% CI: 0.124‐0.538, *P* < .001; Table 5), while such a risk decline was not found in the α‐SMA^low^CD66b^high^, α‐SMA^high^CD66b^low^ or α‐SMA^high^CD66^high^ subgroup patients (Table 5).

**Table 5:** Hazard ratios for DFS and DSS in stage Ⅱ‐III GAC patients receiving adjuvant chemotherapy or not according to α‐SMA and CD66b coexpression patterns

Factor	Patients	%	Adjuvant chemotherapeutic (yes vs no)			
			DFS		DSS	
			HR (95% CI)	*P*‐value	HR (95% CI)	*P*‐value
α‐SMA & CD66b	183		0.482 (0.337‐0.689)	**<.001**	0.476 (0.333‐0.681)	**<.001**
α‐SMA^low^CD66b^low^	65	35.5	0.260 (0.124‐0.542)	**<.001**	0.258 (0.124‐0.538)	**<.001**
α‐SMA^low^CD66b^high^	31	16.9	0.694 (0.298‐1.613)	.396	0.662 (0.285‐1.538)	.337
α‐SMA^high^CD66b^low^	28	15.3	0.736 (0.303‐1.784)	.497	0.694 (0.298‐1.613)	.476
α‐SMA^high^CD66b^high^	59	32.2	0.643 (0.367‐1.125)	.122	0.650 (0.371‐1.139)	.132

Abbreviations: α‐SMA, α‐smooth muscle actin; CI, confidence interval; DFS, disease‐free survival; DSS, disease‐specific survival; GAC, gastric adenocarcinoma; HR, hazard ratio.

*P* < .05 is considered statistically significant (bold).

## 4 DISCUSSION

As a traditional prognostic tool for GAC patients, the TNM classification system is derived from cancer‐centered biological behaviors and overlooks the impacts of the host TME, which result in a significant decline in predictive accuracy. In fact, this is why some patients with early‐phase disease exhibit rapid disease progression, while others with high‐grade disease have stable disease for several years.^19^ In the current research, we detected the immunological parameters CD66b^+^ TANs and α‐SMA^+^ CAFs in 215 GAC specimens with immunohistochemical technology and analyzed the association between these two cell types and their respective and combined prognostic values.

Tumor‐associated neutrophils are a class of sensitized neutrophils in the carcinoma matrix and important components of the TME and play an essential role in tumor evolution; hence, they should be seriously evaluated.^34^ TANs have been considered a compromised prognostic marker in various types of tumors, including bladder cancer, hepatocellular cancer and renal cell cancer.^18^, ^35^, ^36^ The present research demonstrated that a high density of TANs could act as an independent poor prognostic marker in GAC. This is consistent with previous findings.^17^, ^37^ Previous studies have shown that tumor‐infiltrated neutrophils undergo polarization in different TMEs to a procancer N2 or an anticancer N1 subtype.^38^ Therefore, the effects of TANs on tumor cells may be quite varied. Blocking TGF‐β causes a conversion from the procancer phenotype to the anticancer subtype, suggesting that the TAN categorization paradigm resembles the M1/M2‐subtype paradigm of tumor‐associated macrophages.^14^, ^38^ In this study, a high density of TANs in a GAC specimen was associated with a poor clinical outcome and likewise demonstrated that N2‐type neutrophils might be the major cellular phenotype in GAC tissue, despite there being no particular factors that can be applied to discriminate the N1/N2 subsets. Thus, it is necessary to further investigate the precise infiltration profiles of N1 and N2 cells in GAC, their functions and potential biological mechanisms.

In recent years, increasing evidence has shown that the development of carcinoma depends on the intrinsic characteristics of carcinoma cells and the influence of the cancer matrix.^39^ The cancer matrix consists of fibroblasts that generate extracellular matrix (ECM) components, inflammatory cells and blood/lymphatic capillaries.^40^ Sensitized fibroblasts, known as CAFs, have a few similarities with myofibroblasts, including the expression of α‐SMA.^41^ CAFs play an important role in the origination, evolution and diffusion of epithelial carcinoma by generating soluble factors.^42^ They can also reshape the tumor ECM; regulate the metabolism, mobility and stem cell characteristics of carcinoma cells; and prepare disseminated niches.^39^ Previous studies have revealed that CAFs can be a significant prognostic marker in numerous cancers. Ju et al^43^ found that peritumoral CAFs were related to a compromised prognosis in hepatocellular carcinoma patients. Yamashita et al^44^ reported that the expression of α‐SMA was markedly higher in an invasive breast cancer dissemination subgroup than in a no dissemination subgroup and that the invasive subgroup had a worse survival rate. Similarly, Zhi et al^45^ demonstrated that elevated α‐SMA expression was associated with tumor invasiveness characteristics in gastric carcinoma. In the present study, a high density of α‐SMA^+^ CAFs was correlated with the pTNM stage and trends for poor DFS and DSS. In contrast, Valach et al^46^ demonstrated that the extent of α‐SMA expression was not correlated with DFS in head and neck squamous cell carcinoma. The probable cause is that α‐SMA may have diverse expression patterns and biological influences in various types of tumors.

When CD66b^+^ TANs and a‐SMA^+^ CAFs were combined for survival analysis, we discovered that patients with a low density of CD66b^+^ TANs combined with a low density of a‐SMA^+^ CAFs showed the longest DFS and DSS, followed by patients with an a‐SMA^low^CD66b^high^ phenotype, those with an a‐SMA^high^CD66b^low^ phenotype and finally those with an a‐SMA^high^CD66b^high^ phenotype. Multivariate Cox regression revealed that low‐density CD66b^+^ TANs combined with low‐density a‐SMA^+^ CAFs was a good prognostic marker in GAC patients. The combination of the densities of CD66b^+^ TANs and a‐SMA^+^ CAFs with the pathological TNM staging system could better predict the clinical outcome of GAC than the individual parameters. When patients in different pTNM stages were analyzed, the combined predictor still showed potential prognostic value. Neutropenia caused by postoperative chemotherapeutics influences the outcomes of gastric cancer, colon cancer, and breast cancer patients. A short period of neutropenia may indicate a deficient dosage and a lack of lethality.^47^, ^48^, ^49^ Previous studies have shown that the matrix response produces a physical barrier to defend carcinoma cells from chemotherapy in solid tumors,^50^ and inhibiting autophagy in CAFs contributes to the effects of chemotherapy on pancreatic cancer.^51^ Stage II‐III GAC patients with the SMA^low^CD66b^low^ phenotype might benefit from adjuvant chemotherapy. In other words, adjuvant chemotherapy for patients with the a‐SMA^low^CD66b^high^, a‐SMA^high^CD66b^low^ or a‐SMA^high^CD66b^high^ phenotype should be seriously reconsidered. This discovery will help to select more suitable patients for adjuvant chemotherapy treatment and prohibit excessive toxicities and unnecessary resource waste. These conclusions require further, more predictive, multicenter studies for verification.

Molecular biology studies on the interactions between TANs and CAFs are limited. Cheng et al^52^ found that hepatocellular cancer‐derived CAFs affect the survival, activation, and features of neutrophils in hepatocellular cancer via the IL6‐STAT3‐PDL1 signaling pathway. In addition, Zhu et al^53^ suggested that gastric cancer‐derived mesenchymal stem cells protected and activated neutrophils via the IL‐6‐STAT3‐ERK1/2 signaling pathway. In turn, the gastric cancer‐derived mesenchymal stem cell‐primed neutrophils induced the differentiation of normal mesenchymal stem cells into CAFs. Therefore, the cross‐talk between TANs and CAFs is complex, and further research should be conducted on particular mechanisms to determine important targets for antineoplastic therapies.

The limitations of this study are a retrospective study and the relatively small number of patients receiving postoperative chemotherapy. In addition, the small tissues sampled may not represent the entire tumor, which may bias the results. Therefore, a prospective, a multicenter randomized trial is needed to validate these results in the future.

## 5 CONCLUSION

Individually, CD66b^+^ TANs and a‐SMA^+^ CAFs were valuable for predicting prognosis in GAC. However, combining the densities of CD66b^+^ TANs and a‐SMA^+^ CAFs produce a better and more precise prognostic marker that could be applied as a promising prognostic marker for clinical outcomes and indicator for adjuvant chemotherapy treatment decision‐making in GAC patients.

## CONFLICT OF INTERESTS

The authors have declared that no competing interests exist.

## AUTHOR CONTRIBUTIONS

Xiliang Cong contributed to conception, design, data analysis, and writing‐original draft. Yongle Zhang, Ziyu Zhu, and Sen Li contributed to provision of study materials or patients and data analysis and interpretation. Xin Yin, Yu Zhang, and Zhao Zhai contributed to collection and assembly of data; and Yingwei Xue: financial support, technical help, and fruitful discussion.

## ETHICAL APPROVAL

All programs were in accordance with the ethical standards of the Human Subjects Responsibility Committee (institutions and countries) and the 1964 Helsinki Declaration and subsequent versions. This research was approved by the Ethics Committee of Harbin Medical University Cancer Hospital.

## Data Availability

The data that support the findings of this study are available from the corresponding author upon reasonable request.
